# Thoracic Paravertebral Block for Tube Thoracostomy Analgesia in the Emergency Department: A Case Report

**DOI:** 10.5811/cpcem.47070

**Published:** 2025-07-09

**Authors:** M. Townsend Reeves

**Affiliations:** Atrium Health Carolinas Medical Center, Department of Emergency Medicine, Charlotte, North Carolina

**Keywords:** thoracic paravertebral block, tube thoracostomy, pneumothorax, ultrasound-guided regional anesthesia, case report

## Abstract

**Introduction:**

Tube thoracostomy is a common yet exceedingly painful emergency department (ED) procedure, primarily used for pneumothorax evacuation. To facilitate safe performance, stable patients generally receive intravenous anxiolytic or opioid premedication, or perhaps even procedural sedation, in combination with local anesthetic infiltration of the insertion tract. However, more advanced pain management strategies, such as ultrasound-guided truncal blocks, offer a targeted and effective analgesic alternative without the risks or side effect profile inherent to sedation and opioid administration. Herein, a case is presented of painless ED tube thoracostomy following an ultrasound-guided thoracic paravertebral block (TPVB).

**Case Report:**

A 74-year-old female presented to the ED with chest pain and dyspnea from a recurrent, large right-sided spontaneous pneumothorax. An ultrasound-guided thoracic paravertebral block was performed for full-thickness chest wall analgesia prior to tube thoracostomy. A pigtail catheter was inserted painlessly into the pleural space without need for rescue analgesia or procedural sedation, and the pneumothorax was successfully evacuated.

**Conclusion:**

Ultrasound-guided regional anesthesia is increasingly employed in the emergency care setting as part of an opioid-sparing, multimodal analgesia strategy to manage acute pain. For chest tube insertion, the ultrasound-guided thoracic paravertebral block provides potent, long-lasting, and non-euphorigenic, hemithoracic analgesia across multiple contiguous dermatomes from skin to parietal pleura, reducing the need for procedural sedation and opioid therapy while avoiding the incomplete chest wall blockade often associated with other truncal blocks. It is a valuable addition to the analgesic armamentarium of the emergency physician, enabling more comprehensive pain control prior to tube thoracostomy.

## INTRODUCTION

Pneumothorax is the abnormal accumulation of air in the pleural cavity, the potential space between the parietal and visceral pleura lining the inner chest wall and lung, respectively. It is typically classified as traumatic or spontaneous in nature, with the latter further characterized as primary (occurring in absence of underlying lung disease) or secondary (occurring in presence of existing lung disease). Pneumothorax is commonly managed in the emergency department (ED) with tube thoracostomy to evacuate air from the pleural cavity, allowing lung re-expansion and restoration of normal cardiopulmonary function.^1^ While more recent guidelines also support needle aspiration as a less invasive alternative treatment for spontaneous pneumothorax, immediate success rates are lower compared to tube thoracostomy, with the latter more commonly performed.^2^,^3^ Despite its efficacy, tube thoracostomy is a notoriously painful procedure, with 50% of patients reporting severe intraprocedural pain levels of 9–10/10 on a numerical rating scale.^4^

Excluding cases of tension pneumothorax, where expeditious chest tube insertion is prioritized over aggressive pain management to prevent respiratory failure, hemodynamic collapse, and death, emergency physicians traditionally provide intravenous anxiolytic or opioid premedication, combined with local anesthetic infiltration of the insertion tract prior to tube thoracostomy. Some may even use procedural sedation in stable patients needing large-bore (≥ 20 French [F]) chest tube insertion for viscous fluid (pyothorax, hemothorax) evacuation. However, these analgesic strategies are not ideal in many facets. Procedural sedation is time-consuming, resource-intensive, and carries risk of serious complications (eg, apnea, hypoxia, and hypotension), while opioids are habit-forming with a plethora of adverse side effects (eg, nausea/vomiting, pruritus, and delirium). Moreover, insufficient local anesthetic deposition along the chest tube’s tract commonly results in significant insertional and postprocedural pain. The success of local anesthetic infiltration is ultimately predicated on creating a regional field block through large-volume, wide-spread deposition. Unsurprisingly, this method results in significant variability of chest wall anesthesia, with an unpredictable blockade of the intercostal nerve running caudal to each rib between the innermost and internal intercostal muscles. These nerves innervate the parietal pleura, ribs, and intercostal musculature, with their lateral cutaneous branches supplying the overlying skin and subcutaneous tissue of the lateral thorax (Figure).^5^ Intersegmental anastomoses are also common between adjacent intercostal nerves, resulting in variable overlap of classically described dermatomes, further complicating attempts at adequate anesthetic coverage with local infiltration.^6^ Lastly, inadvertent misplacement of the chest tube outside the locally anesthetized tract, even by one intercostal space, will result in block failure.

Ultrasound-guided truncal blocks can provide targeted, reliable, and long-lasting analgesia of the ipsilateral chest wall by blocking hemi-thoracic nerves, either peripherally or at their central paravertebral origins. This makes them a promising analgesic alternative to facilitate safe ED tube thoracostomy. Since the thoracic wall from the superior thoracic aperture to xiphoid process derives its innervation primarily from the spinal nerves of T2–T6, the thoracic paravertebral block (TPVB) is particularly suited out of all truncal blocks to provide complete chest wall analgesia.^5^ By anesthetizing the spinal nerves and dorsal/ventral rami within the thoracic paravertebral space (TPVS), the TPVB inhibits all downstream nociceptive input arising from penetrated chest wall layers (skin, subcutaneous tissue, intercostal muscles, and parietal pleura) (Figure). While the serratus anterior muscle is not anesthetized, the long thoracic nerve (C5–C7) responsible for its innervation is primarily a motor nerve, making its role in pain transmission negligible. In contrast to other truncal blocks (i.e. erector spinae and serratus plane blocks), the TPVB ensures both anterior and posterior hemithorax blockade, as well as reliable anesthesia of the deeper chest wall structures (intercostal musculature, ribs, and parietal pleura) predominantly responsible for the severe pain experienced by patients undergoing tube thoracostomy. Additionally, local anesthetic spread cephalad and caudad within the TPVS ensures multi-level block coverage, allowing for imprecise chest tube delivery above or below the target rib space without risk of block failure.

*CPC-EM Capsule*

What do we already know about this clinical entity?

*Tube thoracostomy is a common yet painful emergency department procedure requiring potent analgesia for safe performance*.

What makes this presentation of disease reportable?

*The thoracic paravertebral block (TPVB) facilitated painless tube thoracostomy in the ED without need for rescue analgesia or procedural sedation*.

What is the major learning point?

*Unlike the serratus and erector spinae plane blocks, the TPVB provides potent, full-thickness analgesia of the anterior and posterior hemithorax across multiple thoracic dermatomes*.

How might this improve emergency medicine practice?

*Emergency physicians who are adept at ultrasound-guided regional anesthesia can safely perform the TPVB for acute thoracic pain conditions*.

Herein, the first case of an emergency physician performed ultrasound-guided TPVB is reported, facilitating painless ED tube thoracostomy for spontaneous pneumothorax evacuation.

## CASE REPORT

A 74-year-old female with past medical history of ovarian cancer with lung metastases, complicated by recurrent right-sided spontaneous pneumothorax, presented to the ED for evaluation of gradually worsening chest pain and dyspnea over the past several days. She reported feeling like her “lung had dropped again.” She was mildly tachypneic though hemodynamically stable with normal oxygen saturation. Physical examination revealed a frail appearing elderly female in mild respiratory distress with absent right-sided breath sounds. A lung point-of-care ultrasound demonstrated absent right-sided lung sliding, and chest radiography confirmed a large right-sided pneumothorax without significant mediastinal or tracheal deviation.

Considering the patient’s history of severe pain from prior tube thoracostomies, and the absence of tension physiology in this case, a tailored analgesic strategy was sought to facilitate pigtail catheter placement. Ultimately, an ultrasound-guided TPVB was performed to provide full-thickness chest wall analgesia prior to catheter insertion (Image, Video).

Informed consent for ultrasound-guided TPVB performance was obtained. The patient was placed on continuous monitoring with intravenous access established. She was positioned sitting upright and a pre-block scan was performed to identify the relevant TPVB sonoanatomy. A 21-gauge, 100-millimeter echogenic block needle was inserted in-plane from lateral-to-medial and into the TPVS apex at the T4 (4th intercostal space) level. The block was completed with 12.5 milliliters (mL) of 0.5% ropivacaine and repeated at the T6 level for a total volume of 25 mL administered. Given the patient weighed 62 kilograms (kg), the total ropivacaine dose (125 milligrams [mg]) was well below the patient’s weight-based maximum (3 mg/kg) for avoidance of local anesthetic systemic toxicity. A total of 10 mg of preservative-free dexamethasone was used as a local anesthetic additive to prolong the block duration.^7^

Approximately thirty minutes post-block performance, the patient reported significantly diminished sensation of the right hemithorax spanning a T2–T7 dermatomal distribution. A lateral approach pigtail tube thoracostomy was performed without need for rescue analgesia or procedural sedation, and the pneumothorax was successfully evacuated. The patient reported feeling pressure but 0/10 intraprocedural pain on a numerical rating scale. She was subsequently admitted for cardiothoracic surgery evaluation. The TPVB lasted approximately 8–10 hours, with the patient reporting minimal (3/10) non-insertion site chest pain managed adequately with a multimodal analgesic regimen during hospitalization. The chest tube was removed on day five, and the patient discharged home in stable condition.

## DISCUSSION

### TPVS Regional Anesthesia Implications

The TPVS runs the length of the thoracic vertebral column bilaterally, and communicates medially with the epidural space and laterally with the intercostal space.^8^,^9^ This allows for multi-segmental, epidural, and intercostal spread of local anesthetic following a single level TPVB injection. By anesthetizing the thoracic spinal nerves/rami and sympathetic chain ganglia, the TPVB provides somatic and sympathetic blockade of the ipsilateral hemithorax across multiple contiguous thoracic dermatomes (Figure).^8^,^9^

### TPVB Performance

While there are numerous variations in ultrasound-guided TPVB technique described in the literature, the choice of approach is generally a matter of proceduralist preference and experience.^8^,^9^ In this case, a transverse-oblique, in-plane approach was performed at the level of the transverse process. Compared to parasagittal approaches, this technique provides the advantage of a shallower needle trajectory and enhanced visualization in relation to the parietal pleura, which may reduce the risk of inadvertent pleural puncture.^8^

Patients can be positioned sitting, lateral decubitus or prone for TPVB performance.^9^ After proper patient positioning, the ribs are counted down posteriorly under dynamic ultrasound guidance till the target injection level is reached. A high-frequency linear array or curvilinear probe (for larger habitus patients) is positioned just lateral to midline in a transverse-oblique orientation, parallel to the underlying rib course. Initially, the hyperechoic transverse process and articulating rib are identified with their confluent acoustic shadows. The probe is then translated slightly caudad into the intercostal space till the TPVS is revealed just lateral to the “thumb-like” contour of the transverse process and between the parietal pleura and internal intercostal membrane (Image).^8^ An 80 – 100-millimeter block needle is inserted in-plane from lateral-to-medial into the apex of the TPVS. Proper needle tip positioning is confirmed by widening of the TPVS and depression (anterior displacement) of the pleural line with test injection (Video).

A multi-level (T4 and T6) ultrasound-guided TPVB was performed using 25 mL of 0.5% ropivacaine with 10 mg dexamethasone as an additive, injecting 12.5 mL per level. However, a clinical study by Uppal et al. demonstrated no difference in dermatomal coverage following a single (T3–T4) versus multiple injection (T1–T5, 5 mL per level) ultrasound-guided TPVB using 25 mL of 0.5% ropivacaine, with both groups resulting in a median of five dermatomes blocked.^10^ Despite being quicker to perform, the single-injection TPVB is more dependent on local anesthetic spread for efficacy, and clinically results in an analgesia level block compared to the surgical anesthesia produced with a multi-injection approach. For ED tube thoracostomy, a single-injection TPVB at the level of chest tube insertion (T4/T5 [4th/5th intercostal space]) is likely sufficient.

### TPVB Contraindications

Major contraindications to TPVB performance include local anesthetic allergy, coagulopathy or systemic anticoagulation, overlying cellulitis, empyema, or paravertebral space-occupying tumor.

### TPVB Potential Complications

Complications after TPVB are relatively uncommon, especially when performed under ultrasound-guidance. Given the proximity of the injection site to the parietal pleura, neuraxis, and intercostal vasculature, potential needle-related complications include pneumothorax (< 1%), nerve damage (< 1%), and vascular puncture (< 1%).^9^ For the purpose of facilitating ED tube thoracostomy, iatrogenic pneumothorax is not of concern when it is already present. Because the TPVS is a non-compressible space, the TPVB should be avoided in patients on anticoagulation to minimize risk of serious bleeding, particularly epidural hematoma. Other reported block complications related to local anesthetic injection/spread include hypotension (from sympathectomy), transient Horner syndrome, and local anesthetic systemic toxicity.^11^ As with all ultrasound-guided nerve blocks, patient monitoring and clear needle tip visualization is paramount to mitigating risk. Emergency physicians must be cognizant of local anesthetic systemic toxicity and adhere to maximum, ideal weight-based dosing of local anesthetic to minimize the occurrence of this potentially fatal clinical entity.

### TPVB Distribution of Anesthesia

Two clinical studies using the transverse-oblique, in-plane approach to the TPVB demonstrated sensory blockade over a median of 4 or 6 dermatomes (range: 3 to 7) following a single 20 mL injection of 0.75% ropivacaine.^12^,^13^ Similarly, a cadaver study observed the spread of 20 mL injected dye over 3 to 4 paravertebral spaces (range: 1 to 10).^14^

### TPVB Limitations

Though generally safe and effective, the ultrasound-guided TPVB is an expert level block by nature of its paravertebral injection location near critical structures. It should not be performed by novices of ultrasound-guided regional anesthesia. Emergency physicians should demonstrate mastery of more readily performed “gateway” nerve blocks (eg, erector spinae and fascia iliaca blocks) prior to attempting TPVB performance. Performance may also be limited in certain patient populations, particularly those with substantial paraspinal tissue or who are morbidly obese, intolerant of repositioning, or needle averse.

### TPVB vs. Other Truncal Blocks

Alternative ultrasound-guided truncal blocks are more commonly performed in the ED for thoracic analgesia. The serratus plane block, by targeting the lateral cutaneous branches of the intercostal nerves, anesthetizes the skin and subcutaneous tissue of the lateral thorax.^15^ However, it does not anesthetize deeper chest wall structures (intercostal musculature, ribs, parietal pleura) involved in tube thoracostomy. In fact, local anesthetic spread has been demonstrated to not reach the intercostal nerves unless concomitant rib fractures are present to disrupt myofascial tissue planes.^16^ In contrast, the erector spinae plane block targets the dorsal rami of the thoracic spinal nerves as they traverse the erector spinae plane. Anterior penetration of local anesthetic into the TPVS does occur though is variable, resulting in unpredictable anterolateral chest wall coverage.^17^ By nature of its proximal paraspinal injection location, the TPVB eliminates the shortcomings of the serratus and erector spinae plane blocks, providing reliable full-thickness coverage across both the anterior and posterior hemithorax.

### Future TPVB ED Applications

Given its comprehensive ipsilateral chest wall coverage, the TPVB has significant analgesic potential for other commonly encountered acute thoracic pain conditions in the ED, such as chest wall burns, herpetic neuralgia, and rib fractures.

## CONCLUSION

Tube thoracostomy is an exceedingly painful ED procedure necessitating effective analgesia to facilitate safe performance. By providing safe and effective hemithorax analgesia or anesthesia, the ultrasound-guided TPVB has potential to reshape established pain management paradigms for tube thoracostomy. Future research should focus on ED utilization of the TPVB for tube thoracostomy in addition to other acute thoracic pain conditions.

## Figures and Tables

**Figure:**
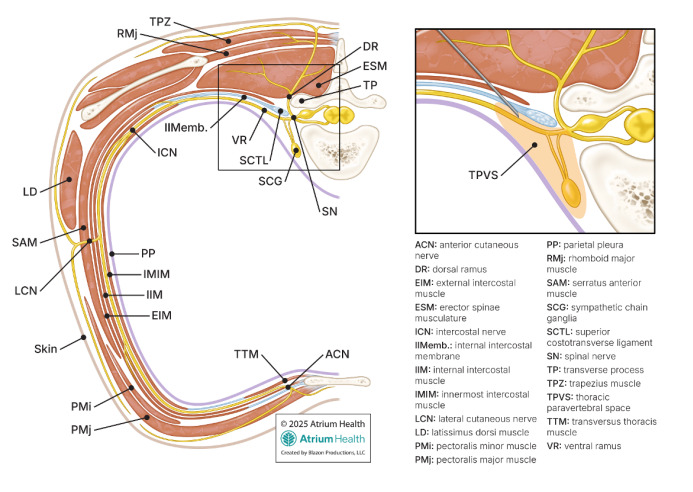
Thoracic wall innervation and paravertebral space anatomy. Each thoracic spinal nerve exits its intervertebral foramen into the paravertebral space, which is bounded medially by the spinal column, anterolaterally by the parietal pleura, and posteriorly by the internal intercostal membrane and superior costotransverse ligament (see inset). Within this wedge-shaped space, spinal nerves communicate with the sympathetic chain anteriorly and divide into dorsal/ventral rami. The ventral rami continue laterally below their corresponding rib as intercostal nerves, initially traveling between the internal intercostal membrane and the parietal pleura, caudal to their accompanying intercostal vein and artery (not shown). Immediately lateral to the angle of the rib, the intercostal nerves and vessels enter the intercostal space between the innermost and internal intercostal muscles for the remainder of their anterior course. Each intercostal nerve sends branches along its trajectory to innervate structures of the anterolateral chest wall including the ribs, intercostal muscles, and parietal pleura. At the midaxillary line, the intercostal nerves give rise to lateral cutaneous branches that pierce the overlying intercostal and serratus anterior muscles to provide sensory innervation to the skin and subcutaneous tissue of the lateral thorax. The intercostal nerves terminate as anterior cutaneous branches that ascend parasternally to innervate the anteromedial chest and midline. In performing an in-plane, transverse-oblique approach to the thoracic paravertebral block, the needle is inserted lateral to medial through the paraspinal musculature, with the tip positioned just anterior to the internal intercostal membrane within the apex of the paravertebral space (see inset).

**Image:**
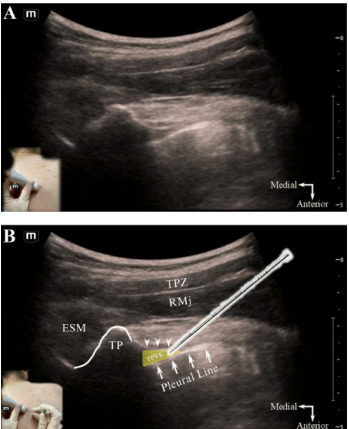
Ultrasound-guided thoracic paravertebral block, in-plane transverse-oblique approach, unlabeled (A) and labeled (B) sonoanatomy. Lateral to the transverse process, the apex of the thoracic paravertebral space (yellow triangle) is seen interposed between the internal intercostal membrane posteriorly (arrowheads) and parietal pleura (arrows) anterolaterally. The block needle is inserted in-plane with the transducer, in a lateral-to-medial direction (see inset, Figure 1B), with the aim of placing the needle tip within the apical part of the thoracic paravertebral space. Abbreviations*: ESM*, erector spinae musculature; *RMj*, rhomboid major muscle; *TP*, transverse process; *TPVS*, thoracic paravertebral space; *TPZ*, trapezius muscle.

**Video:** Ultrasound-guided thoracic paravertebral block performance, in-plane transverse-oblique approach. The block needle tip (arrowhead) is visualized within the apex of the thoracic paravertebral space. Injection confirms proper needle-tip positioning, with expansion of the thoracic paravertebral space and depression (anterior displacement) of the pleural line. *ESM*, erector spinae musculature; *IIMemb*, internal intercostal membrane, *RMj*, rhomboid major muscle; *TP*, transverse process; *TPZ*, trapezius muscle.
